# Laminin-triggered integrin signaling potentiates γ-enolase-induced neurite outgrowth

**DOI:** 10.1007/s11010-026-05755-z

**Published:** 2026-09-30

**Authors:** Selena Horvat, Urša Pečar Fonović, Janko Kos, Anja Pišlar

**Affiliations:** 1 Department of Pharmaceutical Biology, Faculty of Pharmacy, University of Ljubljana, Aškerčeva 7, 1000 Ljubljana, Slovenia; 2 Department of Biotechnology, Jožef Stefan Institute, Jamova 39, 1000 Ljubljana, Slovenia

**Keywords:** γ-Enolase, Laminin, β1 integrin, Signal transduction, Neurite outgrowth

## Abstract

Neurite growth is regulated by trophic support and the extracellular matrix (ECM). γ-Enolase, a neuron-specific enolase isoform, promotes neurite outgrowth through neurotrophic-like activity, but the contribution of ECM engagement and integrin signaling remains unclear. Here, we examined the role of laminin, collagen, and poly-L-lysine in γ-enolase peptide-induced neuritogenesis in neuroblastoma SH-SY5Y and pheochromocytoma PC12 cells. Treatment with a synthetic C-terminal γ-enolase peptide (γ-Eno) rapidly induced neurite outgrowth and actin remodeling, with laminin providing the strongest enhancement, particularly in SH-SY5Y cells. γ-Eno increased surface expression of β1 integrin and, to a lesser extent, α1 integrin, alongside a stronger neurite response on laminin. In laminin-coated cells, actin remodeling was evident within 3 h, followed by increased neurite formation at 24 h and significant neurite elongation at 48 h. Mechanistically, γ-Eno induced focal adhesion kinase (FAK) phosphorylation, which was significantly increased in both cell types on laminin and collagen. Extracellular signal-regulated kinases 1 and 2 (ERK1/2) activation was cell type- and ECM-dependent, whereas Akt phosphorylation increased significantly in SH-SY5Y cells but did not change markedly in PC12 cells. Integrin blockade with GRGDS attenuated γ-Eno-induced neurite outgrowth, MAP2 expression, and FAK phosphorylation. GRGDS also reduced Akt phosphorylation in SH-SY5Y cells and attenuated ERK1/2 phosphorylation in PC12 cells, supporting cell type-specific downstream signaling. These findings show that laminin-mediated integrin signaling enhances γ-enolase-induced neurite outgrowth and is associated with integrin-dependent FAK activation, with ERK1/2 and Akt contributing in a context-dependent manner.

**Supplementary Information:**

The online version contains supplementary material available at https://doi.org/10.1007/s11010-026-05755-z.

## Introduction

Nervous system function depends on highly specific connections formed between neurons during development [1]. In this process, neurotrophic factors play crucial roles in nerve growth and regeneration, cell differentiation, and survival [2, 3]. Among several proteins, neurotrophic activity has also been attributed to the C-terminal region of γ-enolase [4–6].

Enolase is a glycolytic enzyme that can form homo- and heterodimers composed of α, β, and γ subunits [7]. In the mammalian brain, there are three enolase isoforms: αα, αγ, and γγ [8]. The αγ and γγ isoforms, also known as neuron-specific enolase, are primarily found in central nervous system tissues and have been localized only to neurons and neuroendocrine cells [9]. Previous studies have shown that the intact C-terminal region of neuron-specific enolase exerts neurotrophic activity when bound to the plasma membrane of neuronal cells [5, 6, 10, 11]. The scaffold protein γ1-syntrophin participates in γ-enolase translocation to the plasma membrane, enabling its neurotrophic activity. Once translocated, γ-enolase promotes neuronal survival and neurite outgrowth by activating phosphatidylinositol 3-kinase (PI3K) and mitogen-activated protein kinase (MAPK) signal transduction pathways, resulting in downstream regulation of molecular and cellular processes such as cytoskeleton reorganization and cell remodeling, activation of transcription factors, and regulation of the cell cycle [4, 12].

Cell adhesion to extracellular matrix (ECM) proteins regulates key cellular functions, including cell-cycle progression, proliferation, and differentiation [13, 14]. In the nervous system, ECM proteins contribute to neuronal development by regulating neural progenitor behavior, neuronal migration, neurite outgrowth, axon elongation, and synapse formation, largely through interactions with integrin receptors [15, 16]. Integrins are a major family of transmembrane adhesion receptors that link ECM components to the cytoskeleton and transduce biochemical and mechanical signals between cells and their microenvironment [17, 18]. They consist of α and β subunits that form heterodimeric receptors, with different subunit combinations conferring specificity for distinct ECM ligands [18]. For example, the integrin α1β1 binds to specific domains of the laminin molecule, promoting neurite outgrowth [19]. Interactions between ECM molecules and integrins activate multiple signaling pathways that overlap with growth factor and cytokine signaling [16, 18]. Among the key molecules activated in integrin-mediated signaling are focal adhesion kinase (FAK) and Src-family kinases. These kinases are recruited to sites of integrin-mediated adhesion along with other structural proteins, leading to morphological changes that support processes such as cell spreading, growth, survival and migration [20, 21].

In this study, we investigated how the extracellular matrix context and integrin-associated signaling contribute to the neuritogenic effects of γ-enolase. Our findings show that laminin enhances γ-enolase-induced neurite outgrowth, actin polymerization, and cytoskeletal remodeling. γ-Eno treatment also increased cell-surface expression of β1 integrin and activated FAK and MAPK/ERK signaling. Moreover, attenuation of the neuritogenic response by GRGDS supports a contribution of RGD-binding integrins.

## Materials and methods

### Materials

A synthetic peptide corresponding to the C-terminal 30-amino-acid sequence of human brain γ-enolase (γ-Eno; AKYNQLMRIEEELGDEARFAGHNFRNPSVL) was synthesized by Biosynthesis (Lewisville, TX, USA). Laminin (L2020), collagen (C3867), poly-L-lysine (P4707) and the RGD-containing integrin antagonist GRGDS (G4391), were obtained from Sigma (St. Louis, MO, USA).

### Cell culture

Human neuroblastoma SH-SY5Y cells (ATCC CRL-2266; RRID: CVCL_0019) and rat pheochromocytoma PC12 cells (ATCC CRL-1721; RRID: CVCL_0481) were obtained directly from the American Type Culture Collection (ATCC, Manassas, VA, USA) in 2010 and used between passages 20 and 30. SH-SY5Y cells were maintained in Advanced Dulbecco’s Modified Eagle Medium (Thermo Fisher Scientific, Waltham, MA, USA) supplemented with 10% fetal bovine serum (FBS; Thermo Fisher Scientific), 1% L-glutamine (G7513; Sigma-Aldrich), and 1% penicillin-streptomycin (P4333; Sigma-Aldrich). PC12 cells were cultured in Advanced Dulbecco’s Modified Eagle Medium supplemented with 5% fetal bovine serum, 10% heat-inactivated horse serum (HS; 26050088; Thermo Fisher Scientific), 1% L-glutamine, and 1% penicillin-streptomycin. Cells were maintained at 37 °C in a humidified incubator with 5% CO₂ and passaged at approximately 80% confluence. The experimental cell stocks were not independently authenticated in our laboratory; however, all experimental groups were compared using the same ATCC-derived stocks under identical culture conditions. SH-SY5Y cells were confirmed to be free of mycoplasma contamination in 2022, whereas PC12 cells were not independently tested for mycoplasma.

### Coating protocol and cell treatment

Plates were coated with collagen (10 µg/mL), laminin (10 µg/mL), or poly-L-lysine (10 µg/mL) diluted in Dulbecco’s phosphate-buffered saline (DPBS; D8537, Sigma-Aldrich), incubated at 37 °C for 2 h, and then washed with DPBS before seeding the cells. Cells cultured on pre-coated plates were treated the next day with the synthetic peptide γ-Eno at a concentration of 100 nM in serum-free or reduced medium for the indicated times. When required, cultured cells were pre-treated with GRGDS peptide at a concentration of 10 µM for 1 h prior to γ-Eno peptide (100 nM) treatment.

### Cellular metabolic activity

Cellular metabolic activity was assessed using the MTS assay. SH-SY5Y and PC12 cells were seeded in pre-coated 96-well culture plates in complete growth medium at a density of 2 × 10⁴ cells/mL, with four technical replicates per condition. The following day, cells were treated as described above. At the indicated time points, MTS signal was determined using the CellTiter 96^®^ AQueous One Solution Cell Proliferation Assay (Promega, WI, USA), according to the manufacturer’s instructions. Absorbance was measured at 490 nm using an automated microplate reader (Tecan Safire^2^, Switzerland). Results are expressed as percentages relative to untreated control cells.

### Cell proliferation assay

Cell proliferation was evaluated using carboxyfluorescein succinimidyl ester (CFSE) labeling followed by flow cytometric analysis. SH-SY5Y and PC12 cells were seeded in pre-coated 12-well culture plates in complete growth medium at a density of 5 × 10⁴ cells/mL, with two technical replicates per condition. The following day, cells were labeled with 1 µM CellTrace™ CFSE reagent (C34554; Thermo Fisher Scientific) according to the manufacturer’s instructions. After staining, cells were treated as described above. Cells were subsequently collected, and CFSE fluorescence intensity was measured in the BL-1 channel using an Attune NxT flow cytometer (Thermo Fisher Scientific). Data were analyzed using FlowJo software, and mean CFSE fluorescence intensity was normalized to that of untreated control cells.

### Real-time impedance analysis

Cellular responses were monitored using a Real-Time Cell Analyzer Dual Plate (RTCA DP) Instrument with the xCELLigence System (Agilent). E plates were coated with collagen, laminin, and poly-L-lysine as indicated. After washing with DPBS, SH-SY5Y and PC12 cells were seeded at a concentration 1 ⋅ 10^4^ cells/well in serum-free medium, in presence or absence of γ-Eno peptide (100 nM). Electrical impedance at the electrode surface was recorded every 10 min for 24 h and converted by the RTCA software into a dimensionless cell index (CI), reflecting changes in cell-electrode interactions. The area under the curve (AUC) was calculated from the CI–time profiles as an integrated measure of the impedance-derived cellular response over time.

### Evaluation of neurite outgrowth

Neurite outgrowth was evaluated after 24 and 48 h of treatment using phase-contrast images acquired with an Olympus IX81 motorized inverted microscope (Olympus Biosystems GmbH, Munich, Germany) and the EVOS Cell Imaging System (Thermo Fisher Scientific). At least 100 cells per condition were evaluated for both analyses in each independent biological experiment. Cells bearing one or more neurite-like processes with a length at least twice the diameter of the cell body were classified as neurite-positive. The proportion of neurite-bearing cells was calculated and expressed relative to the corresponding untreated control for each substrate. Neurite length was additionally quantified from the same phase-contrast images using ImageJ. Neurite-like processes were traced from the edge of the cell body to the distal tip. Mean neurite length was calculated for each condition, normalized to the corresponding untreated control, and expressed as fold change.

### Confocal microscopy analysis

For fluorescence staining, SH-SY5Y and PC12 cells were seeded in duplicate onto pre-coated glass coverslips placed in 24-well culture plates at a density of 2 × 10⁴ cells/mL in growth medium. Following treatment, the culture medium was removed, and cells were fixed with 4% paraformaldehyde (PFA; 1510; Electron Microscopy Sciences) in DPBS, with or without Ca²⁺ and Mg²⁺, for 20 min at room temperature. Cells were washed with phosphate-buffered saline (PBS). For phalloidin staining, cells were permeabilized with 0.1% Triton X-100 (T8787; Sigma-Aldrich) in PBS, pH 7.4, for 3 min at room temperature. For GAP-43 immunostaining, cells were permeabilized with 0.5% Tween-20 (P9416; Sigma-Aldrich) in PBS for 10 min. Non-specific binding was blocked with 10% donkey serum (S-30; Sigma-Aldrich) in PBS containing 0.05% Triton X-100 for 30 min at room temperature. For neuronal marker staining, cells were incubated with rabbit anti-GAP-43 antibody (1:150; ab75810; Abcam) diluted in blocking solution for 2 h at room temperature, followed by incubation with Alexa Fluor 555-conjugated secondary antibody (1:1000; Thermo Fisher Scientific) for 1.5 h at room temperature. For visualization of filamentous actin, cells were stained with TRITC-conjugated phalloidin (500 ng/mL; P1951; Sigma-Aldrich) in 20 mM Tris-HCl buffer, pH 7.4, for 30 min at 37 °C. After staining, cells were washed three times with PBS for 5 min each. Nuclei were counterstained and coverslips were mounted using ProLong™ Gold Antifade Reagent with DAPI (P36935; Thermo Fisher Scientific). Fluorescence images were acquired using a Nikon AX confocal microscope (Nikon, Tokyo, Japan) and processed with NIS-Elements software (Nikon). TRITC-phalloidin and GAP-43 fluorescence intensities were quantified using ImageJ. Images were separated into their respective fluorescence channels, and regions containing the scale bar were excluded from analysis. Background fluorescence was corrected using the Subtract Background function with a rolling-ball radius of 25 pixels. Mean fluorescence intensity was determined over the full field of view. Identical image-acquisition and analysis settings were applied to all experimental groups.

### Western blotting

For cell lysate preparation, SH-SY5Y and PC12 cells were seeded into precoated 6-well plates at a density of 7.5 × 10^5^ cells/mL and treated as described above. Cells were then harvested, washed with PBS, and lysed using lysis buffer (150 mM NaCl, pH 6.5, 50 mM HEPES, 1 mM EDTA, and 0.25% Triton X-100). The lysates were then preserved at − 80 °C to undergo freeze-thaw cycles. For final cell lysate preparation, cells were sonicated and centrifuged at 15,000 rpm for 15 min at 4 °C. The protein concentration in the obtained supernatants was quantified using a DC Protein Assay kit (Bio-Rad, CA, USA). Bovine serum albumin was used as a protein standard to determine the protein concentrations of the samples. Equal concentrations of protein from cell lysates (1 µg/µL) were denatured by the addition of SDS-PAGE buffer, heated for 5 min, and resolved by SDS-PAGE on 12% Tris-glycine gels. The proteins were then transferred to PVDF membranes using iBlot (Thermo Fisher Scientific). Membranes were blocked in 5% (w/v) non-fat dried milk powder in Tris-buffered saline with Tween 20 (TBST; 20 mM Tris/HCl, 137 mM NaCl, pH 7.4, and 0.1% Tween 20) at RT for 1 h. Subsequently, the membranes were incubated overnight at 4 °C with primary antibodies in TBST containing 3% (w/v) bovine serum albumin (A2153; Sigma-Aldrich). Primary antibodies included rabbit anti-phospho-Akt (ph-Akt [Ser^473^], 1:1000, 9271, Cell Signaling), rabbit anti Akt (1:1000, 9272, Cell Signaling), rabbit anti phospho-ERK1/2 (ph-ERK1/2 [Thr^202^/Tyr^204^], 1;1000, 9101 S, Cell Signaling), rabbit anti ERK1 (1:1000, sc-93, Santa Cruz Biotechnology, CA, USA), rabbit anti ERK2 (1;1000, sc-154, Santa Cruz Biotechnology), and rabbit anti- glyceraldehyde-3-phosphate dehydrogenase (GAPDH; 1:10000; 10494-1-AP; Proteintech). After washing, secondary horseradish peroxidase-conjugated anti-rabbit antibody (1:5000; 111-035-045; Jackson ImmunoResearch) was added in TBST containing 5% (w/v) non-fat dried milk powder for 1 h. After washing, protein bands were visualized with enhanced chemiluminescence detection kits (34075 or 34095; Thermo Fisher Scientific) and recorded with a G: Box imager (Syngene, UK). When necessary, membranes were stripped with buffer (100 mM 2-mercaptoethanol, 2% SDS, and 62.5 mM Tris/HCl, pH 5.7) for 1 h at 65 °C.

### Flow cytometry analysis of phosphorylated proteins

SH-SY5Y and PC12 cells were seeded into pre-coated 24-well plates at a density of 2 × 10⁵ cells/mL and treated as described above. After treatment, cells were harvested, washed with PBS, and fixed with 5% formalin (HT501128; Sigma-Aldrich) for 10 min at room temperature. Cells were then permeabilized with ice-cold methanol for 20 min at 4 °C. Non-specific staining was blocked with 3% BSA (A1253; Sigma-Aldrich) in PBS for 30 min. Cells were subsequently incubated with primary antibodies against phospho-Akt (ph-Akt [Ser^473^]; 9271; Cell Signaling Technology, Danvers, MA, USA), phospho-FAK (ph-FAK [Tyr^397^]; sc-11765; Santa Cruz Biotechnology), or phospho-ERK1/2 (ph-ERK1/2 [Thr^202^/Tyr^204^]; 9101 S; Cell Signaling Technology) diluted in blocking buffer according to the manufacturers’ recommendations for 45 min at 4 °C. After washing, cells were incubated with Alexa Fluor 488-conjugated secondary antibodies (1:1000; Thermo Fisher Scientific) for 30 min at room temperature protected from light. Finally, cells were washed twice with PBS, and fluorescence intensity was measured in the BL-1 channel using an Attune NxT flow cytometer (Thermo Fisher Scientific). Data were analyzed using FlowJo software, and mean fluorescence intensity was normalized to that of untreated control cells.

### Flow cytometry for surface expression of integrin subunits

SH-SY5Y and PC12 cells were seeded into pre-coated 24-well plates at a density of 2 × 10⁵ cells/mL and treated as described above. At the indicated time points, cells were washed with ice-cold PBS, followed by washing with 3% BSA in PBS containing 1% sodium azide. Non-specific staining was blocked with 3% BSA in PBS. Cells were then incubated with primary antibodies against the α1-integrin subunit (sc-271034; Santa Cruz Biotechnology), β1-integrin subunit (sc-8978; Santa Cruz Biotechnology), or β2-integrin subunit (sc-6624; Santa Cruz Biotechnology), each at a concentration of 4 µg/mL, for 45 min at 4 °C. Cells were subsequently incubated with Alexa Fluor 555-conjugated secondary antibodies (1:500; Thermo Fisher Scientific) for 45 min at room temperature. After staining, cells were washed and fixed with 5% formalin. Mean fluorescence intensity was measured using an Attune NxT flow cytometer (Thermo Fisher Scientific). Data were analyzed using FlowJo software. Alexa Fluor 555-conjugated IgG was used as an isotype control.

### Flow cytometry analysis of MAP2 expression

SH-SY5Y and PC12 cells were seeded into pre-coated 24-well culture plates at a density of 5 × 10⁴ cells/mL and treated as described above. At the indicated time points, cells were washed with ice-cold PBS, fixed with 5% formalin for 15 min at room temperature, and permeabilized with ice-cold methanol for 20 min at 4 °C. Non-specific staining was blocked with 3% BSA in PBS for 30 min. Cells were then incubated with rabbit anti-MAP2 antibody (ab32454; Abcam) diluted in blocking buffer for 45 min at 4 °C. After washing, cells were incubated with Alexa Fluor 555-conjugated secondary antibody (1:500; Thermo Fisher Scientific) for 30 min at room temperature protected from light. Cells were subsequently collected, and mean fluorescence intensity was measured using an Attune NxT flow cytometer (Thermo Fisher Scientific). Data were analyzed using FlowJo software. Alexa Fluor 555-conjugated IgG was used as an isotype control.

### Statistical analysis

Data are presented as mean ± standard error of the mean (SEM). The number of independent experiments and technical replicates is indicated in the corresponding figure legends. Statistical significance was assessed using one-way analysis of variance (ANOVA), followed by Tukey’s post hoc test for multiple comparisons. Analyses were performed using GraphPad Prism version 10. Differences were considered statistically significant at *P* < 0.05.

## Results

### γ-Enolase-treated cells show increased neurite response on laminin-coated surface

**Fig. 1:**
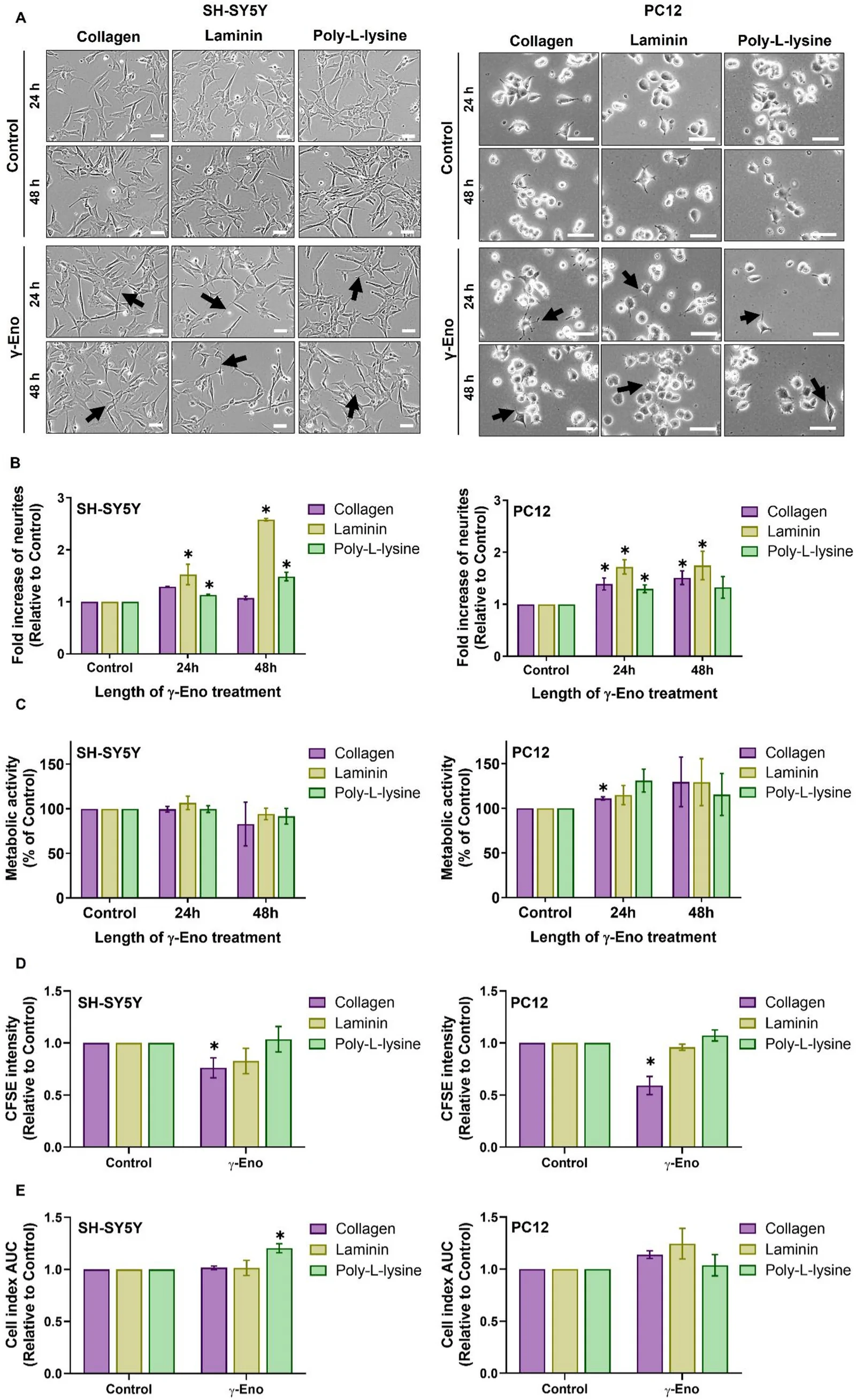
Effects of γ-Eno peptide on cellular responses to various extracellular matrix proteins. SH-SY5Y (*left*) and PC12 cells (*right*) were cultured on plates coated with collagen, laminin, or poly-L-lysine (10 µg/mL) and treated with γ-enolase peptide (γ-Eno, 100 nM) for the indicated times. **A** Representative phase-contrast images of cells after 24 h and 48 h of treatment. Black arrows indicate neurite-like processes. Scale bars: 50 μm. **B** Neurite outgrowth was quantified after 24 h and 48 h of treatment. Cells bearing at least one neurite twice the diameter of the cell body were scored as neurite-positive (*N* = 3 per cell line). **C** Cellular metabolic activity was assessed by MTS assay after 24 h and 48 h of treatment (SH-SY5Y: *N* = 2, PC12: *N* = 3, each performed in quadruplicate). **D** Cell proliferation was assessed after 24 h with carboxyfluorescein succinimidyl ester (CFSE) labeling and flow cytometry (*N* = 3 per cell line, each performed in duplicate). **E** The impedance-derived cell-index response was monitored using the xCELLigence System (*N* = 4 per cell line, each performed in quadruplicate). Data are expressed relative to the control and presented as mean ± SEM. Statistical significance was assessed by one-way ANOVA followed by Tukey’s multiple-comparisons test; *p < 0.05

Previous work showed that a peptide corresponding to the C-terminal region of γ-enolase (γ-Eno) promotes neurite outgrowth and implicated an association with tropomyosin receptor kinase (Trk)-related signaling [4, 22]. In the present study, we further explored whether engagement with the extracellular matrix (ECM) modifies the neuritogenic response to γ-Eno through integrin-associated signaling. SH-SY5Y and PC12 cells were cultured on laminin-, collagen-, or poly-L-lysine-coated surfaces in serum-free medium and treated with γ-Eno (100 nM). Following treatment, both cell types developed neuritic processes (Fig. 1A). Quantification of neurite-bearing cells revealed a time-, cell type-, and substrate-dependent response (Fig. 1B). In SH-SY5Y cells, the proportion of neurite-bearing cells was significantly increased on laminin and poly-L-lysine after both 24 and 48 h, with the largest relative response observed on laminin. In PC12 cells, the proportion of neurite-bearing cells was increased on all tested substrates after 24 h and remained elevated mainly on collagen and laminin after 48 h. Direct measurement of neurite length provided complementary evidence of neurite elongation. γ-Eno significantly increased mean neurite length on laminin after 48 h in both SH-SY5Y and PC12 cells, whereas changes on collagen and poly-L-lysine did not reach statistical significance (Supplementary Fig. S1). To ensure that the observed morphological changes were accompanied by alterations in cellular metabolic activity, we performed an MTS assay following γ-Eno peptide treatment for 24 and 48 h across all tested ECM substrates. The results showed no significant reduction in MTS signal in either SH-SY5Y or PC12 cells at both time points, indicating that γ-Eno did not adversely affect cellular metabolic activity under the tested conditions (Fig. 1C). Moreover, a slight upward trend in MTS signal was observed in PC12 cells, suggesting enhanced metabolic activity in a substrate-dependent cellular context; however, this trend was not statistically significant. To further assess whether γ-Eno influences cell proliferation, we performed a CFSE assay after 24 h of treatment. A significant reduction in CFSE fluorescence intensity was observed in both SH-SY5Y and PC12 cells cultured on collagen-coated surfaces, indicating increased cell proliferation under these conditions (Fig. 1D). In contrast, no significant changes in CFSE intensity were detected on laminin- or poly-L-lysine-coated surfaces, suggesting that the proliferative effect of γ-Eno is most pronounced in the presence of collagen. We next investigated whether γ-Eno altered the impedance-derived cellular response measured using the xCELLigence system. γ-Eno significantly increased the cell index in SH-SY5Y cells cultured on poly-L-lysine (Fig. 1E). In contrast, no significant changes in cell index were observed in PC12 cells across all tested ECM substrates following γ-Eno treatment.

**Fig. 2:**
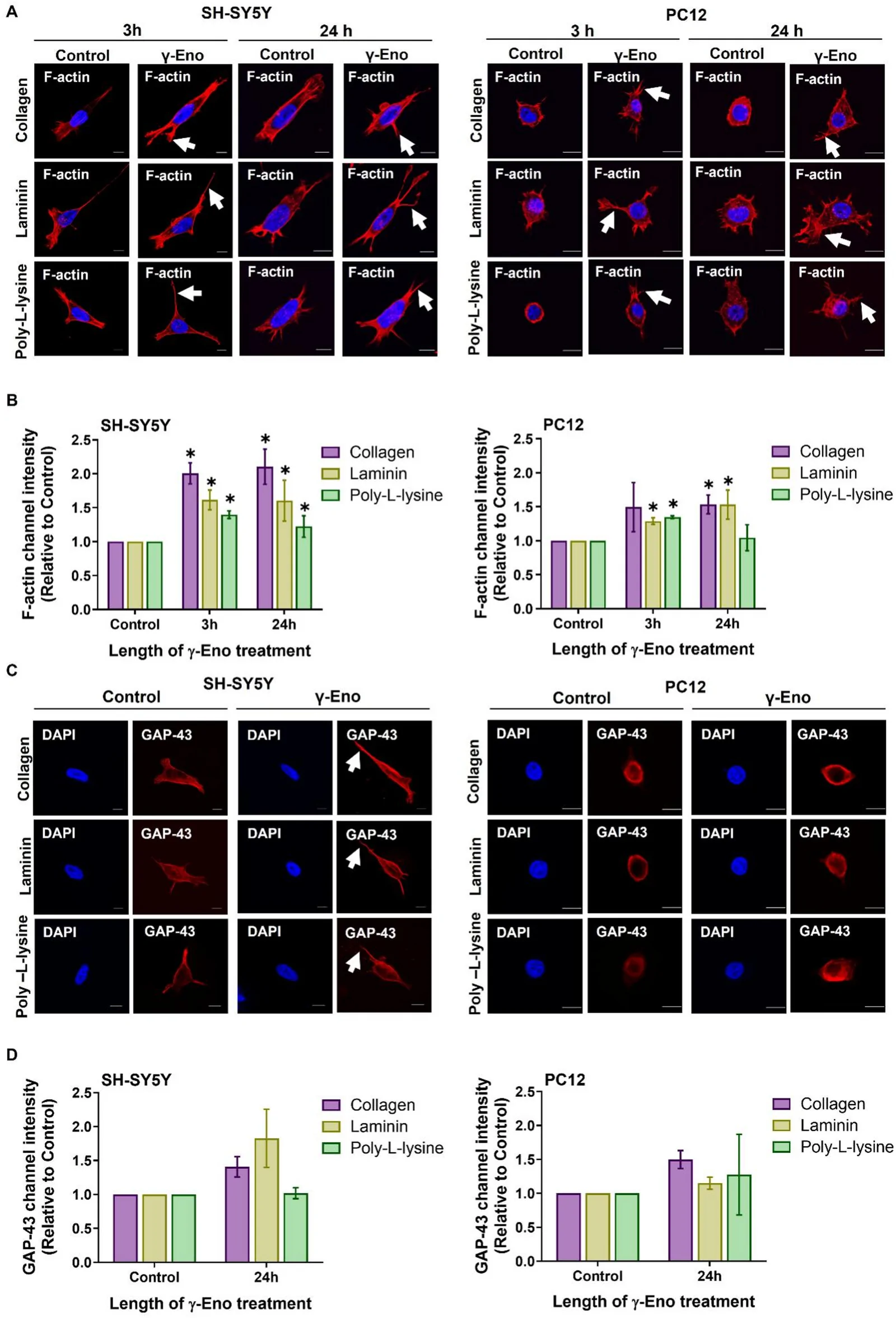
Effects of γ-Eno peptide on cytoskeletal remodeling in response to various extracellular matrix proteins. SH-SY5Y (*left*) and PC12 cells (*right*) were cultured on plates coated with collagen, laminin, or poly-L-lysine (10 µg/mL) and treated with γ-enolase peptide (γ-Eno, 100 nM) for the indicated times. **A** Representative fluorescence images of F-actin stained with TRITC-conjugated phalloidin (*red*) after 3–24 h of γ-Eno treatment. **B** Quantification of F-actin fluorescence intensity expressed relative to the corresponding untreated control (3 h: *N* = 2 per cell line, 24 h: *N* = 4 per cell line). **C** Representative fluorescence images of GAP-43 immunostaining (*red*) after 24 h of treatment. **D** Quantification of GAP-43 fluorescence intensity expressed relative to the corresponding untreated control (*N* = 2 per cell line). Nuclei were counterstained with DAPI (*blue*), and white arrows indicate neurite-like processes. Scale bars: 10 μm. Data are expressed relative to the control and presented as mean ± SEM. Statistical significance was assessed by one-way ANOVA followed by Tukey’s multiple-comparisons test; *p < 0.05

To further investigate the cytoskeletal dynamics underlying γ-Eno-induced neurite outgrowth, we performed F-actin staining using TRITC-conjugated phalloidin. Representative images showed γ-Eno-associated changes in cell spreading and the formation of neurite-like processes in both SH-SY5Y and PC12 cells after 3 and 24 h of treatment across the different coating conditions (Fig. 2A). Quantification of F-actin fluorescence intensity revealed a cell line-, time-, and substrate-dependent response (Fig. 2B). In SH-SY5Y cells, the strongest increase was observed on collagen after 3 h, and F-actin intensity remained elevated after 24 h. More moderate increases were detected on laminin and poly-L-lysine. In PC12 cells, γ-Eno induced smaller increases in F-actin intensity, with the greatest response observed on laminin after 24 h. In addition to F-actin remodeling, neurite outgrowth was further supported by GAP-43 immunostaining after 24 h of γ-Eno treatment (Fig. 2C). In SH-SY5Y cells, γ-Eno increased GAP-43 fluorescence intensity on collagen and, more prominently, on laminin, whereas GAP-43 intensity remained close to control levels on poly-L-lysine (Fig. 2D). Representative images also showed more prominent GAP-43-positive neurite-like processes in γ-Eno-treated SH-SY5Y cells. In PC12 cells, changes in GAP-43 intensity were more modest and variable, with the largest increase observed on collagen and only limited changes on laminin and poly-L-lysine. Consistent with these quantitative findings, PC12 cells did not exhibit the same degree of pronounced neurite-like elongation observed in SH-SY5Y cells. Together, these results indicate that γ-Eno promotes cytoskeletal remodeling and neurite-associated GAP-43 expression in a substrate- and cell line-dependent manner, with a more pronounced neuritogenic response in SH-SY5Y cells.

### Pronounced activation of signaling pathways in response to γ-eno and laminin

**Fig. 3:**
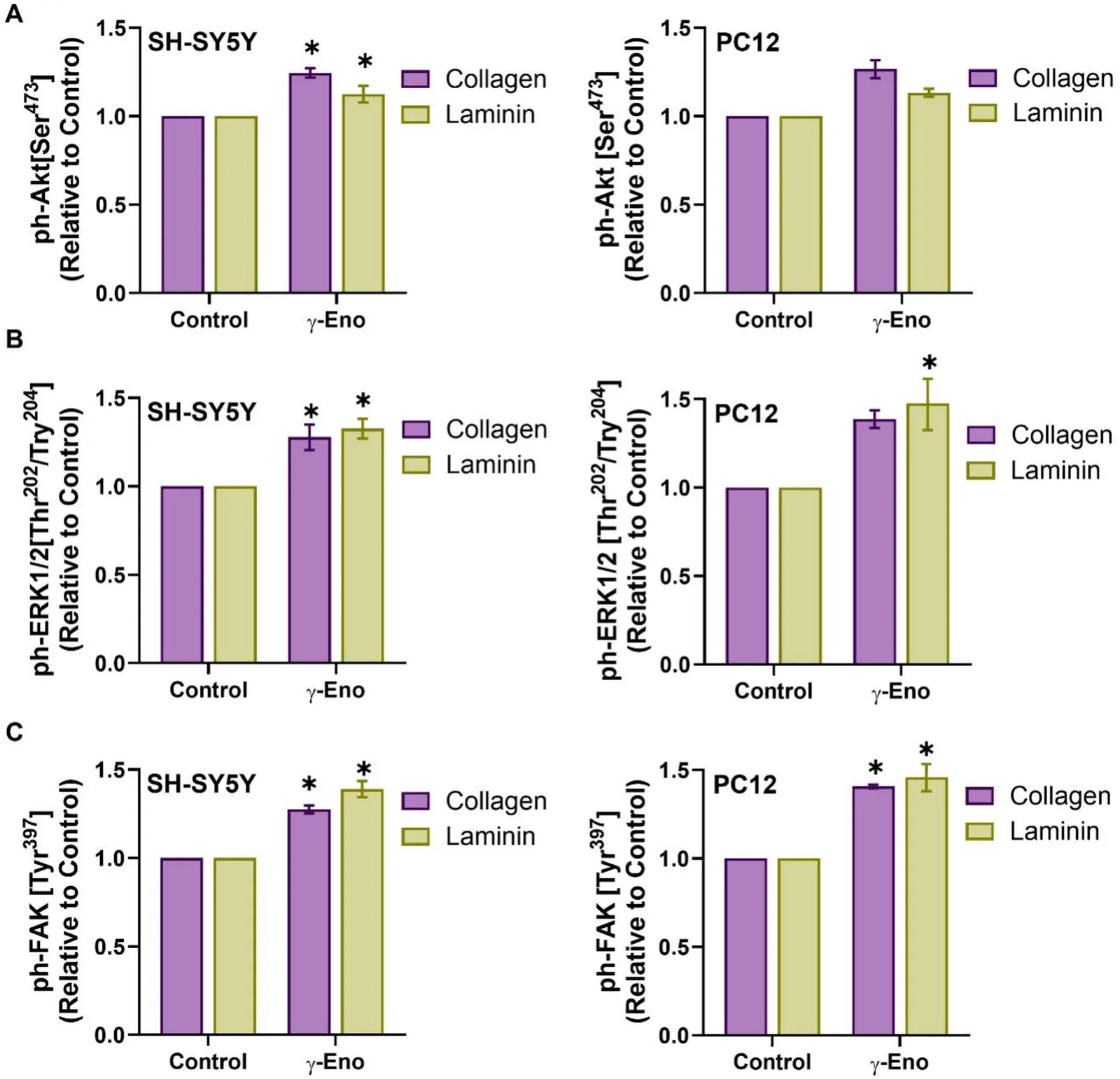
γ-Eno peptide induces distinct signaling responses depending on extracellular matrix proteins. **A**–**C** SH-SY5Y (*left*) and PC12 (*right*) cells were cultured on plates coated with collagen or laminin (10 µg/mL) and treated with γ-enolase peptide (γ-Eno, 100 nM) for 24 h. Flow cytometric analyses were performed to assess **A** Akt using an antibody specific for ph-Akt [Ser^473^] (SH-SY5Y: collagen, *N* = 4; laminin, *N* = 8; PC12: collagen, *N* = 4; laminin, *N* = 5), **B**) extracellular signal regulated kinase 1/2 (ERK1/2) using an antibody specific for ph-ERK1/2 [Thr^202^/Tyr ^204^] (SH-SY5Y: collagen, *N* = 5; laminin, *N* = 4; PC12: collagen and laminin, *N* = 4 each), and **C** focal adhesion kinase (FAK) using an antibody specific for ph-FAK [Tyr^397^] (SH-SY5Y: collagen, *N* = 2; laminin, *N* = 3; PC12: collagen, *N* = 8; laminin, *N* = 4). Each independent experiment was performed in duplicate. Data are expressed relative to the control and presented as mean ± SEM. Statistical significance was assessed by one-way ANOVA followed by Tukey’s multiple-comparisons test; *p < 0.05

To further elucidate the intracellular signaling mechanisms underlying γ-Eno-induced neurite outgrowth, we investigated the activation of key downstream signaling pathways, including Akt, MAPK/ERK, and FAK, in response to 24 h γ-Eno treatment on collagen and laminin. In SH-SY5Y cells, phosphorylation of Akt at Ser473 was significantly increased by γ-Eno on both collagen and laminin, whereas in PC12 cells only a modest, non-significant increase was observed under both conditions (Fig. 3A). These findings suggest that Akt signaling may contribute to the γ-Eno response in SH-SY5Y cells but appears to play a less prominent role in PC12 cells. Phosphorylation of ERK1/2 (Thr202/Tyr204) was significantly increased by γ-Eno in SH-SY5Y cells on both collagen and laminin (Fig. 3B). In PC12 cells, ERK1/2 phosphorylation was significantly increased on laminin, while only a non-significant upward trend was observed on collagen (Fig. 3B). These findings regarding Akt and ERK1/2 phosphorylation were supported by western blot analysis in both SH-SY5Y and PC12 cells (Supplementary Fig. S2). Given the established role of integrins in activating FAK and related scaffolding proteins [23], we next examined the activation status of FAK. Flow cytometric analysis revealed that γ-Eno significantly increased phosphorylation of FAK at Tyr397 in both SH-SY5Y and PC12 cells cultured on either collagen or laminin (Fig. 3C). Taken together, these results indicate that γ-Eno activates neuritogenic signaling through both ERK1/2 and FAK pathways, while Akt activation appears to be cell type-dependent. Laminin tended to support stronger signaling responses, particularly for ERK1/2 activation in PC12 cells, although γ-Eno-induced FAK activation was evident on both ECM substrates.

### Upregulation of β1 integrin subunit surface expression by γ-Eno

**Fig. 4:**
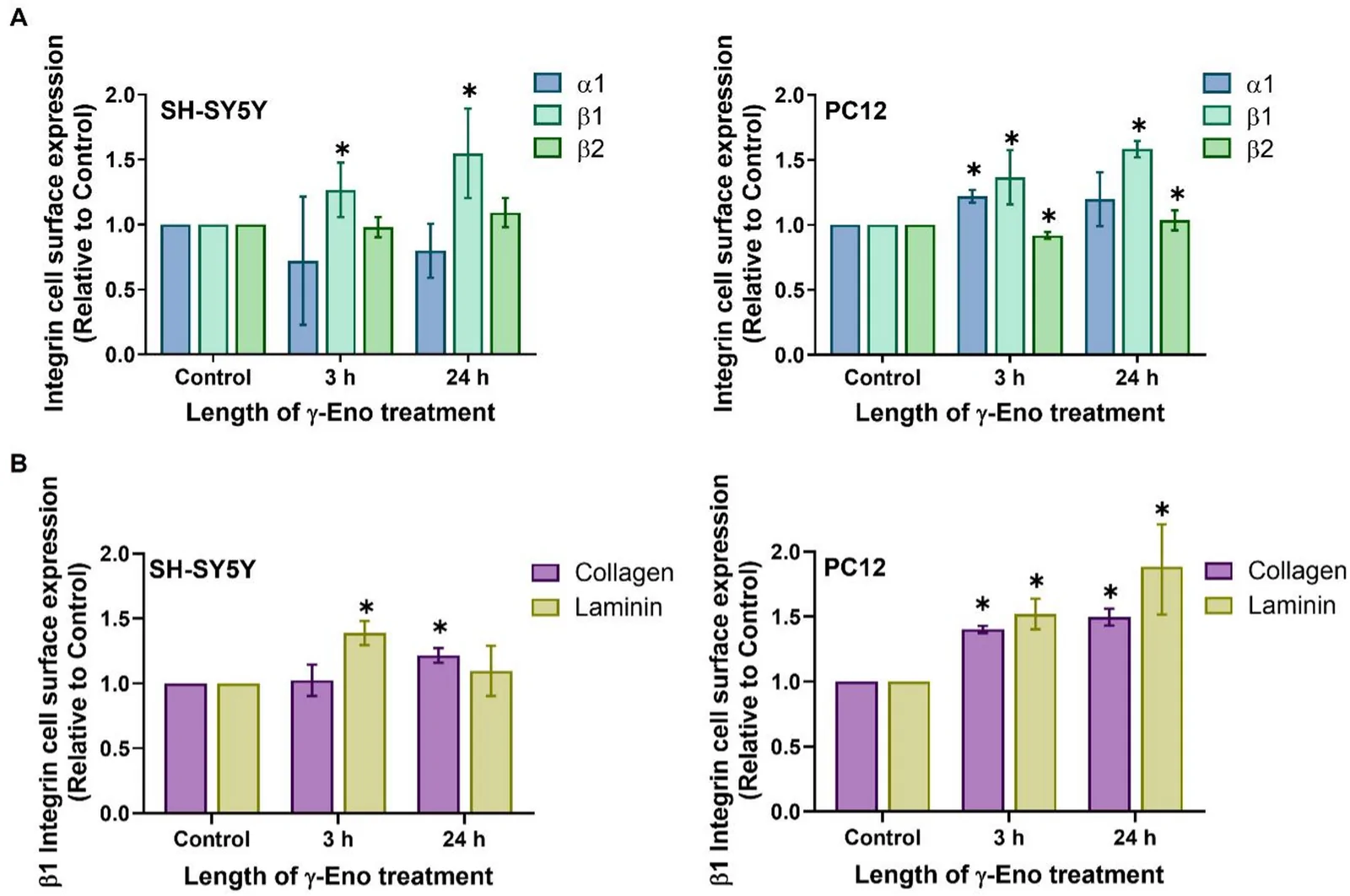
Effects of γ-Eno peptide on the cell-surface expression of selected integrin subunits in SH-SY5Y and PC12 cells. **A** SH-SY5Y (*left*) and PC12 cells (*right*) were cultured on uncoated plates and treated the following day with γ-enolase peptide (γ-Eno, 100 nM) for 3–24 h. Cell-surface expression of α1-, β1-, and β2-integrin subunits was analyzed by flow cytometry in non-permeabilized cells (SH-SY5Y: α1, *N* = 2; β1, *N* = 4; β2, *N* = 4; PC12: α1, *N* = 2; β1, *N* = 4; β2, *N* = 2). **B** Cells were cultured on plates coated with collagen or laminin (10 µg/mL), treated with γ-Eno for 3–24 h, and analysed for cell-surface expression of the β1-integrin subunit (SH-SY5Y: collagen, *N* = 3; laminin, *N* = 2; PC12: both coatings, *N* = 4). Each independent experiment was performed in duplicate. Data are expressed relative to the control and presented as mean ± SEM. Statistical significance was assessed by one-way ANOVA followed by Tukey’s multiple-comparisons test; *p < 0.05

Cells adhere to extracellular matrix (ECM) proteins through integrin receptors, some of which play key roles in neurite outgrowth and neuronal differentiation [24, 25]. To determine whether γ-enolase modulates integrin expression, we examined the surface levels of α1, β1, and β2 integrin subunits in SH-SY5Y and PC12 cells using flow cytometry. The α1 and β1 subunits were selected because they form the α1β1 heterodimer, a well-characterized laminin receptor previously implicated in neurite outgrowth in SH-SY5Y and PC12 cells [26]. The β2 subunit was included as a comparison because it is not among the established laminin-binding integrin subunits [27]. On uncoated plastic surfaces, β1 integrin subunit expression significantly increased 3 h after γ-Eno treatment in both cell lines, with an even more pronounced increase at 24 h (Fig. 4A). A similar rapid increase was observed for the α1 subunit in PC12 cells, reaching significance at 3 h, although this effect did not persist at 24 h. In contrast, β2 integrin subunit surface expression did not increase in either cell line following γ-Eno treatment. Notably, β2 expression was significantly downregulated in PC12 cells at both time points. To further explore β1 integrin expression in the context of ECM interaction, cells were cultured on laminin- and collagen-coated surfaces. In SH-SY5Y cells, γ-Eno treatment led to a marked increase in β1 integrin surface expression after 3 h on laminin, while no significant change was observed on collagen (Fig. 4B). However, after 24 h, β1 expression decreased on laminin and significantly increased on collagen. In contrast, PC12 cells showed a consistent and significant increase in β1 integrin expression on both laminin and collagen at 3 h, with an even greater effect at 24 h. At both time points, laminin supported the highest levels of β1 integrin surface expression in PC12 cells. Among the integrin subunits examined, β1 showed the most consistent γ-Eno-induced increase, with a time- and ECM-dependent expression pattern that was particularly pronounced on laminin.

### Antagonism of RGD-binding integrins reduces γ-enolase-mediated neuritogenic effect

**Fig. 5:**
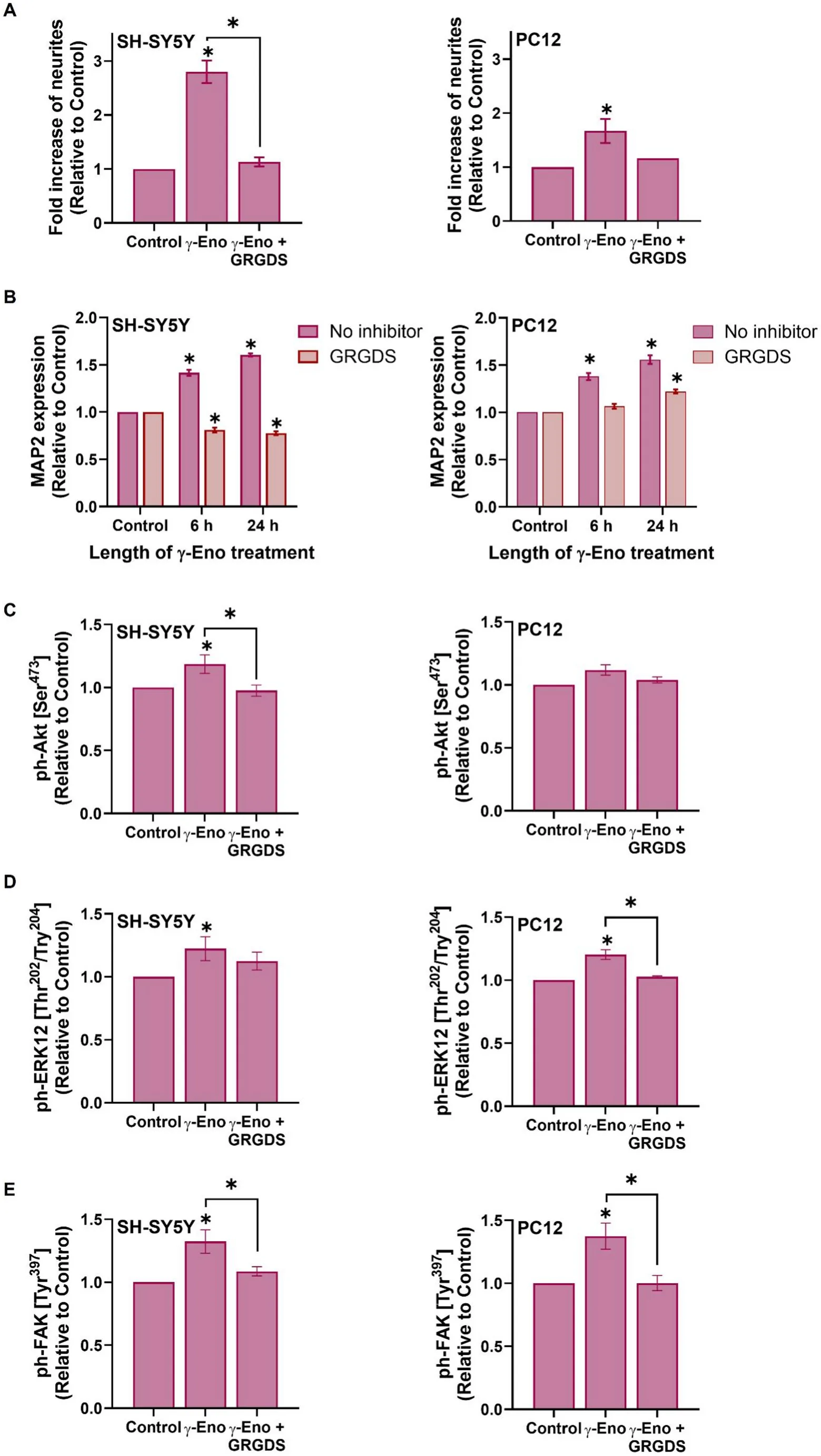
GRGDS-mediated antagonism of RGD-binding integrins reduces the γ-Eno peptide-mediated neuritogenic effect. SH-SY5Y (*left*) and PC12 cells (*right*) were cultured on laminin-coated plates (10 µg/mL) and treated with γ-enolase peptide (γ-Eno, 100 nM) in the absence or presence of GRGDS peptide (10 µM) for the indicated times. **A** Neurite outgrowth was quantified after 24 h. Cells bearing at least one neurite twice the diameter of the cell body were scored as neurite-positive (*N* = 4 per cell line). **B**–**E** Flow cytometric analyses were performed to assess expression of **B** microtubule-associated protein 2 (MAP2; *N* = 2 per cell line), activation of **C** Akt using an antibody specific for ph-Akt [Ser^473^] (SH-SY5Y, *N* = 6; PC12, *N* = 3), **D** extracellular signal regulated kinase 1/2 (ERK1/2) using an antibody specific for ph-ERK1/2 [Thr^202^/Tyr ^204^] (*N* = 4 per cell line), and **E** focal adhesion kinase (FAK) using an antibody specific for ph-FAK [Tyr^397^] (SH-SY5Y, *N* = 4; PC12, *N* = 5). Each independent experiment was performed in duplicate. Data are expressed relative to the control and presented as mean ± SEM. Statistical significance was assessed by one-way ANOVA followed by Tukey’s multiple-comparisons test; * *p* < 0.05

To evaluate the contribution of RGD-binding integrins to γ-enolase-promoted neurite outgrowth, SH-SY5Y and PC12 cells were pre-treated with a synthetic GRGDS peptide. GRGDS can competitively inhibit ligand interactions with several RGD-binding integrins and therefore does not distinguish the involvement of individual integrin subunits or heterodimers [28, 29]. Cells cultured on laminin-coated surfaces were incubated with 10 µM GRGDS peptide for 1 h prior to γ-Eno treatment. This pre-treatment significantly inhibited γ-Eno-induced neurite formation in SH-SY5Y cells and attenuated the response in PC12 cells, reducing neurite-bearing cells to near-control levels (Fig. 5A). Direct neurite-length analysis confirmed that γ-Eno significantly increased mean neurite length on laminin and that GRGDS significantly attenuated this increase in both SH-SY5Y and PC12 cells (Supplementary Fig. S3). To further assess the effect of integrin inhibition on neuronal differentiation markers, we examined MAP2 expression in SH-SY5Y and PC12 cells. Cells cultured on laminin and treated with γ-Eno showed a significant upregulation of MAP2 protein at both 6 and 24 h in both cell lines. However, this increase was markedly attenuated in the presence of the GRGDS peptide; in SH-SY5Y cells, MAP2 levels decreased below control values at both time points, whereas in PC12 cells MAP2 expression remained close to control at 6 h and was only modestly elevated at 24 h (Fig. 5B), indicating that integrin engagement is required for the full induction of MAP2 in response to γ-Eno. Integrin blockade also affected downstream signaling. In SH-SY5Y cells, GRGDS significantly reduced γ-Eno-induced Akt phosphorylation, whereas no significant changes in Akt phosphorylation were observed in PC12 cells (Fig. 5C). For ERK1/2, GRGDS did not significantly suppress the γ-Eno-induced response in SH-SY5Y cells, but significantly reduced ERK1/2 phosphorylation in PC12 cells (Fig. 5D). Moreover, γ-Eno treatment significantly increased phosphorylation of FAK in both cell lines cultured on laminin, and GRGDS pre-treatment significantly reduced FAK phosphorylation to near-control levels in both cell lines (Fig. 5E). Collectively, these findings support a contribution of RGD-sensitive integrin signaling to γ-Eno-induced neurite outgrowth and neuronal differentiation. FAK was the most consistently affected integrin-associated signaling molecule, whereas the effects on Akt and ERK1/2 were cell type-dependent.

## Discussion

Our findings demonstrate that γ-Eno-induced neurite outgrowth is strongly influenced by the extracellular matrix and is most effectively potentiated by laminin. In both SH-SY5Y and PC12 cells, γ-Eno increased β1 integrin surface expression and enhanced FAK phosphorylation, supporting the involvement of integrin-associated signaling in the γ-Eno response. ERK1/2 activation was dependent on both cell type and ECM substrate, whereas Akt activation was more pronounced in SH-SY5Y cells. Together, these results support the involvement of laminin-associated integrin signaling, with FAK as a prominent downstream component, as an important modulator of the neuritogenic response to γ-enolase.

Neurite outgrowth is regarded as a consequence of the differentiation of precursor cells into a mature neuronal phenotype [30]. SH-SY5Y and PC12 cells are widely used in vitro models for studying neuronal differentiation in response to neurotrophic stimulation [31, 32]. Previous reports have shown that the neurotrophic-like factor γ-enolase enhances neurite outgrowth through activation of the PI3K/Akt and MAPK/ERK signaling pathways [4], which are also commonly engaged by established neurotrophic factors. These factors typically activate intracellular signaling through Trk receptors [3, 33], and γ-enolase has been reported to associate with the Trk signaling complex in differentiated SH-SY5Y cells [22]. Functional interplay between Trk and integrin signaling has been implicated in regulating neurotrophic factor-mediated cellular responses [20, 25]. Accordingly, ECM components can modulate neuronal differentiation and neurite regeneration in combination with neurotrophic factors and differentiation-inducing agents [34, 35]. Within this framework, our findings support a role for ECM-dependent integrin signaling in modulating the cellular response to γ-Eno and are consistent with a potential interaction with neurotrophin receptor-associated pathways. As Trk activation was not directly evaluated in the present study, its precise contribution to this response remains to be established.

In line with this, our findings show that the ECM component laminin enhances γ-Eno-induced differentiation of SH-SY5Y and PC12 cells into neuron-like cells characterized by pronounced neurite formation. These results are consistent with previous studies reporting that ECM proteins potentiate growth factor-induced neuronal differentiation [20, 36]. We observed that laminin enhanced γ-Eno peptide-induced actin polymerization, supporting rapid cytoskeletal remodeling and rapid neurite formation [37, 38]. Among the ECM proteins tested, laminin most effectively potentiated neurite outgrowth. F-actin staining revealed pronounced morphological changes as rapid as 3 h after γ-Eno peptide treatment. Quantification of neurite-bearing cells demonstrated an early neuritogenic response within 24 h, whereas direct measurement of neurite length showed significant elongation on laminin after 48 h in both cell lines. These complementary findings suggest that γ-Eno initially increases the proportion of responding cells and subsequently promotes neurite elongation in a substrate-dependent manner.

The collagen-specific increase in cell proliferation, indicated by greater CFSE dye dilution, appears to be separable from the neuritogenic response. Laminin supported the strongest neurite formation without a detectable increase in proliferation, whereas collagen increased proliferation but did not consistently produce the strongest neurite response. This distinction is consistent with previous studies showing that laminin strongly supports neurite outgrowth in SH-SY5Y cells and that neuronal differentiation and neurite-like processes in PC12 cells can be accompanied by reduced or arrested proliferation [34, 39]. Moreover, extracellular matrix proteins have been shown to differentially regulate the proliferation and differentiation of PC12 cells [40]. Collectively, these findings suggest that γ-Eno may influence the balance between proliferation and differentiation in an ECM-dependent manner, rather than increased proliferation directly driving neurite formation. Likewise, the increased impedance-derived cell index observed in SH-SY5Y cells on poly-L-lysine does not necessarily indicate enhanced neuritogenic signaling. The cell index reflects changes in electrical impedance associated with cell–electrode interactions, whereas poly-L-lysine primarily supports nonspecific electrostatic cell attachment and laminin can engage specific cell-surface receptors and activate biochemical signaling [41, 42]. Thus, an increased impedance-derived cellular response and the ability of a substrate to support integrin-associated neuritogenic signaling represent distinct cellular properties.

Consistent with the established role of laminin in promoting neuronal differentiation via integrin-associated FAK-MAPK signaling, our findings further show that γ-enolase facilitates neuritogenic signaling in an ECM-dependent manner. γ-Eno treatment significantly increased FAK phosphorylation in both SH-SY5Y and PC12 cells on both laminin and collagen, indicating that FAK is a consistent downstream effector of the γ-Eno response. ERK1/2 phosphorylation was also increased by γ-Eno, but the pattern differed between the two cell lines: SH-SY5Y cells showed significant ERK1/2 activation on both collagen and laminin, whereas PC12 cells showed a clear increase primarily on laminin, with only a weaker response on collagen. Akt phosphorylation showed an additional cell type-specific pattern, being significantly increased in SH-SY5Y cells but not markedly altered in PC12 cells. The distinct Akt responses may reflect differences between the two cellular models, including their species and tissue of origin, differentiation state, and broader signaling context [39]. PI3K/Akt signaling has been implicated in neuronal differentiation of SH-SY5Y cells [43], whereas Ras/ERK signaling is well established as an important component of differentiation in PC12 cells [44]. FAK-family kinases may also contribute by integrating growth factor- and integrin-derived signals during neurite outgrowth [20]. These differences could therefore partly account for the more pronounced Akt response observed in SH-SY5Y cells, while PC12 cells may depend relatively more on FAK/ERK-associated signaling. This aligns with previous studies showing that laminin-integrin interactions enhance ERK signaling during neurite outgrowth in various neuronal models, including PC12 cells and mesenchymal stem cells [42]. Taken together, these findings support a model in which γ-enolase promotes neurite signaling mainly through FAK, with ERK1/2 as an important downstream pathway and Akt contributing in a more limited, cell type-dependent manner.

To better understand the molecular basis of γ-Eno-enhanced neurite outgrowth on laminin, we examined the expression of integrin subunits involved in ECM adhesion. Our findings revealed increased levels of the β1 integrin subunit, and to a lesser extent, the α1 integrin subunit on the cell surface of SH-SY5Y and PC12 cells in response to γ-Eno treatment. β2 integrin surface expression was not increased by γ-Eno; instead, it was significantly reduced in PC12 cells at both time points. The increased β1 integrin surface expression may contribute to the enhanced neuritogenic response observed on laminin. As reported, a similar effect was observed with the differentiation agent retinoic acid, which induced specific upregulation of the laminin-binding integrin α1β1 complex in SH-SY5Y cells [26]. Additionally, increased expression of α1β1 integrin was also observed in PC12 cells after treatment with NGF, correlating with increased neurite response to laminin [36, 45]. Thus, the increased expression of β1-containing integrins, potentially including α1β1, may represent one mechanism contributing to enhanced neurite-like processes on laminin. However, laminin-dependent neuronal responses can also be mediated by other integrins, including α3β1, α6β1, and α7β1 [19, 46, 47]. Because these receptors were not examined in the present study, their contribution to the γ-Eno response cannot be excluded.

Integrin activation can trigger several distinct intracellular pathways that appear to use the same intermediates as neurotrophic factor signaling through tyrosine kinase receptors. Although FAK is an important component of integrin-associated signaling, it functions within a broader network of kinases and adaptor proteins [48]. FAK is activated by integrin engagement and can interact either directly or through adaptor proteins such as talin or paxillin with the cytoplasmic tail of integrin β subunits [25]. In addition to activating FAK, some β1 and α integrins also activate the tyrosine kinase Fyn and, through it, the adaptor protein Shc [49]. Both FAK and Src have been implicated in axonal growth and neurite-like processes in response to integrin activation [25, 50–52]. Src-family kinases can cooperate with FAK in downstream signaling and cytoskeletal remodeling, whereas paxillin can coordinate signaling complexes at sites of integrin engagement [20, 21, 25, 50, 51]. Integrin-linked kinase (ILK) may provide an additional link between integrin engagement, Akt signaling, and neurite outgrowth [53]. Thus, although FAK phosphorylation was the most consistent integrin-associated signaling response measured in our study, Src-family kinases, paxillin, ILK, and other integrin-associated proteins may also contribute to the observed phenotype. We observed a significant increase in FAK activation after exposure to γ-Eno peptide. Phosphorylation of FAK increased in γ-Eno-treated cells on both laminin and collagen, rather than being restricted to laminin alone. However, FAK phosphorylation was assessed by flow cytometry and was not independently validated by western blotting. FAK may contribute to activation of the MAPK/ERK cascade when Src-linked integrins bind to ECM components [54]. In our culture system, ERK1/2 activation was enhanced by γ-Eno in both cell lines, although the laminin-dependent effect was more pronounced in PC12 cells.

Use of the RGD-containing integrin antagonist GRGDS provided additional functional support for the contribution of RGD-binding integrins to γ-Eno-induced neurite outgrowth. The RGD motif is recognized by several integrin heterodimers, and soluble RGD-containing peptides can competitively interfere with these ligand-integrin interactions [28, 29]. GRGDS attenuated γ-Eno-induced neurite outgrowth and reduced MAP2 expression in both SH-SY5Y and PC12 cells. The inhibitory effect of GRGDS was further supported by direct neurite-length measurements, which showed significant attenuation of γ-Eno-induced neurite elongation in both cell lines. These findings support the involvement of signaling associated with RGD-binding integrins but do not identify the specific integrin heterodimer responsible for the response. Although the observed increase in β1 integrin surface expression supports β1-containing integrins as plausible contributors, other RGD-binding integrins may also participate. Establishing a specific functional requirement for β1 integrin will require selective approaches, such as β1-blocking antibodies or β1 knockdown. Moreover, GRGDS pre-treatment significantly reduced γ-Eno-induced FAK phosphorylation in both cell lines, reduced Akt phosphorylation in SH-SY5Y cells, and attenuated ERK1/2 phosphorylation in PC12 cells. These results indicate that integrin engagement contributes to the full γ-enolase signaling, although the downstream pathway usage differs between the two cell lines. These findings are consistent with previous studies showing that disruption of integrin-FAK interactions can impair neurite outgrowth, highlighting the importance of integrin-mediated adhesion signaling in neuronal development [20, 42].

**Fig. 6:**
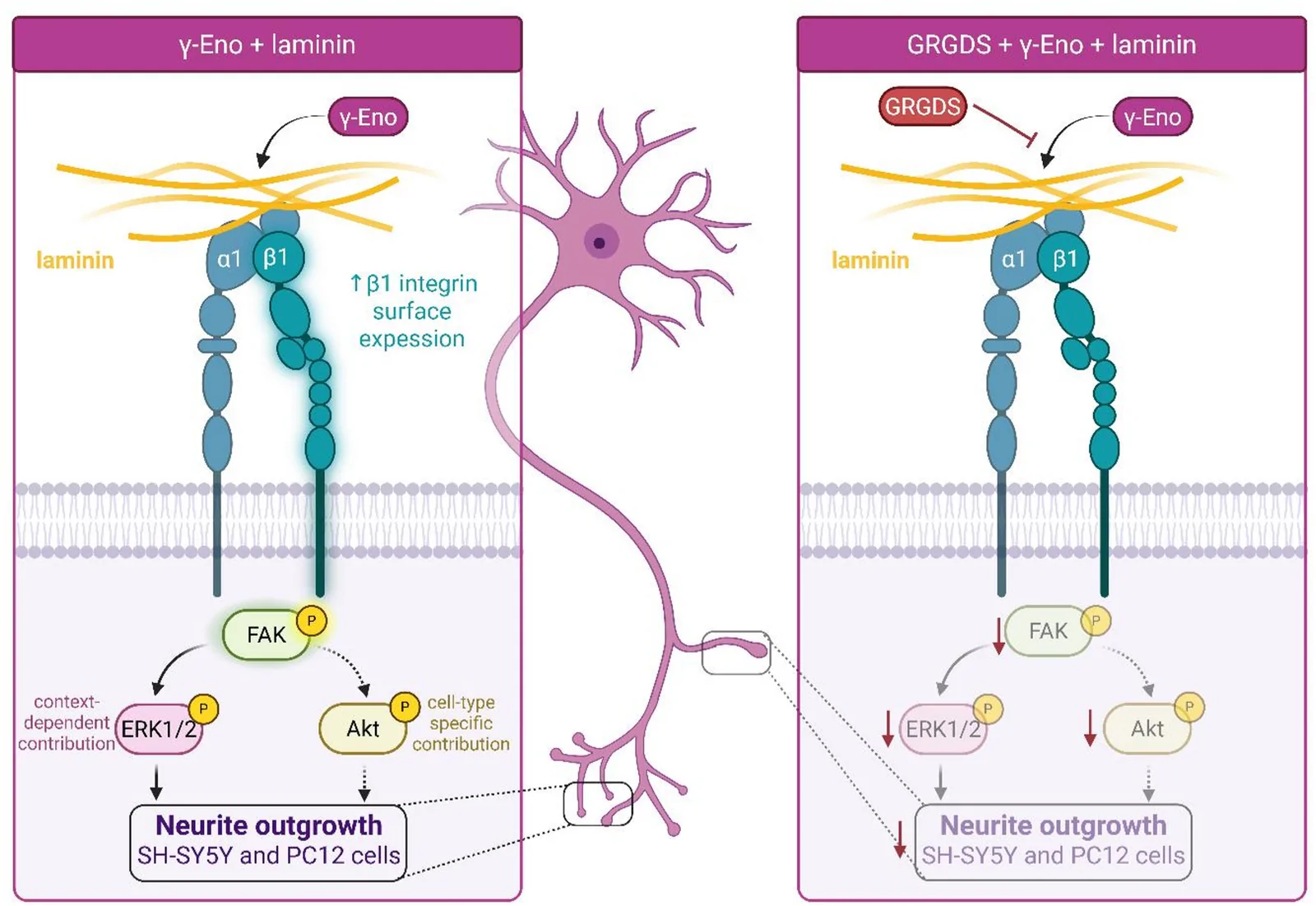
Effect of GRGDS on γ-Eno-induced laminin-associated integrin signaling and neurite outgrowth. Schematic representation of γ-Eno-mediated neurite outgrowth on laminin and its inhibition by GRGDS in SH-SY5Y and PC12 cells. γ-Eno increases β1 integrin surface expression and promotes laminin-associated integrin signaling, leading to FAK activation and cell type-dependent modulation of ERK1/2 and Akt signaling. GRGDS attenuates γ-Eno-induced downstream signaling and neurite outgrowth. Created with BioRender

In conclusion, the findings of this study indicate that integrin engagement enhances the neuritogenic effect of the γ-Eno peptide in neuronal-like SH-SY5Y and PC12 cells and is associated with robust FAK activation and context-dependent ERK1/2 signaling, whereas Akt activation appears to be more cell type-specific (Fig. 6). Although FAK was the most consistently activated integrin-associated molecule examined, additional downstream components, including Src-family kinases, paxillin, and ILK, may also contribute to the observed response. Furthermore, γ-Eno-induced changes in β1 integrin and, to a lesser extent, α1 integrin expression were associated with enhanced neurite outgrowth in cells cultured on laminin. Together with the GRGDS-mediated attenuation of the neuritogenic response, these findings support a contribution of RGD-sensitive integrin signaling to the effects of γ-Eno, although the specific integrin heterodimer involved remains to be established. These findings suggest a potential mechanism through which γ-enolase may modulate ECM-dependent neuritogenic signaling, with possible relevance to neuronal differentiation and regeneration.

## Supplementary Information

Below is the link to the electronic supplementary material.

Supplementary Material 1

## Data Availability

The datasets generated and/or analyzed during the current study are available from the corresponding author on reasonable request.
