# Supplementary material for: Laminin-triggered integrin signaling potentiates γ-enolase-induced neurite outgrowth
Source: Mol Cell Biochem. 2026 Sep 30;481(10):5745–60. doi: 10.1007/s11010-026-05755-z (PMC13687352; doi:10.1007/s11010-026-05755-z)
Supplement: Supplementary file 1 [file 11010_2026_5755_MOESM1_ESM.docx]

**Laminin-triggered integrin signaling potentiates γ-enolase-induced neurite outgrowth**

Selena Horvat ([selena.horvat@ffa.uni-lj.si](mailto:selena.horvat@ffa.uni-lj.si))^a^, Urša Pečar Fonović ([ursa.pecarfonovic@ffa.uni-lj.si)^a^](mailto:ursa.pecarfonovic@ffa.uni-lj.si)a), Janko Kos ([janko.kos@ffa.uni-lj.si](mailto:janko.kos@ffa.uni-lj.si))^a,b^, Anja Pišlar ([anja.pislar@ffa.uni-lj.si)^a,*^](mailto:anja.pislar@ffa.uni-lj.si)a,*)

^a^ Department of Pharmaceutical Biology, Faculty of Pharmacy, University of Ljubljana, Aškerčeva 7, 1000 Ljubljana, Slovenia

^b^ Department of Biotechnology, Jožef Stefan Institute, Jamova 39, 1000 Ljubljana, Slovenia

**Supplemental Material**

**Fig. S1.** **Neurite length in γ-Eno-treated SH-SY5Y and PC12 cells cultured on different substrates.** SH-SY5Y (**A**) and PC12 (**B**) cells were cultured on plates pre-coated with collagen, laminin, or poly-L-lysine (10 μg/mL) and treated with γ-enolase peptide (γ-Eno, 100 nM) for 24 or 48 h. Neurite length was quantified from phase-contrast images using. Neurite length was determined relative to the corresponding untreated control for each substrate. Data are presented as mean ± SEM from three (N=3) independent biological experiments. Statistical significance was assessed using one-way ANOVA followed by Tukey’s multiple-comparisons test; **p* < 0.05 versus the corresponding untreated control**.**

**Fig. S2. Western blot analysis of Akt and ERK1/2 phosphorylation in γ-Eno-treated SH-SY5Y and PC12 cells.** SH-SY5Y and PC12 cells were cultured on plates pre-coated with collagen or laminin (10 μg/mL) and treated with γ-enolase peptide (γ-Eno, 100 nM) for 24 h. (**A** - **D**) Representative Western blots and densitometric quantification of Akt phosphorylation at Ser473 are shown for SH-SY5Y (**A**) and PC12 (**B**) cells, whereas ERK1/2 phosphorylation at Thr202/Tyr204 is shown for SH-SY5Y (**C**) and PC12 (**D**) cells. Phosphorylated Akt and ERK1/2 levels were normalized to the corresponding total Akt and total ERK1/2 levels, respectively, and expressed relative to the corresponding untreated control for each substrate. GAPDH was used as a loading control. Data are presented as mean ± SEM from N =3 independent biological experiments. Statistical significance was assessed using one-way ANOVA followed by Tukey’s post hoc test; **p* < 0.05 versus the corresponding untreated control.

**Fig. S3.** **Effect of GRGDS on γ-Eno-induced neurite elongation in SH-SY5Y and PC12 cells.** SH-SY5Y (**A**) and PC12 (**B**) cells were cultured on plates pre-coated with laminin (10 μg/mL) and treated with γ-enolase peptide (γ-Eno, 100 nM) for 24 h in the absence or presence of GRGDS (10 μM). Cells were pre-treated with GRGDS for 1 h before γ-Eno exposure. Neurite length was quantified from phase-contrast images using ImageJ. Mean neurite length was expressed relative to the untreated control. Data are presented as mean ± SEM from three (N=3) independent biological experiments. Statistical significance was assessed using one-way ANOVA followed by Tukey’s multiple-comparisons test; **P* < 0.05 versus the untreated control and versus γ-Eno alone.
